# Effectiveness of structured patient-clinician communication with a solution focused approach (DIALOG+) in community treatment of patients with psychosis – a cluster randomised controlled trial

**DOI:** 10.1186/1471-244X-13-173

**Published:** 2013-06-26

**Authors:** Stefan Priebe, Lauren Kelley, Eoin Golden, Paul McCrone, David Kingdon, Clare Rutterford, Rosemarie McCabe

**Affiliations:** 1 Unit for Social and Community Psychiatry, Queen Mary University of London, Newham Centre for Mental Health, London E13 8SP, UK; 2 Kings College London, Health Service and Population Research Department, Institute of Psychiatry, De Crespigny Park, London, SE, UK; 3 Faculty of Medicine, University of Southampton, Building 85, Life Sciences Building, Highfield Campus, Southampton SO17 1BJ, UK; 4 Centre for Primary Care and Public Health, Blizard Institute, Barts and The London School of Medicine and Dentistry, Yvonne Carter Building, 58 Turner Street, London E1 3AB, UK

## Abstract

**Background:**

Large numbers of patients with psychosis have regular meetings with key clinicians in the community. There is little evidence on how these meetings should be conducted to be therapeutically effective. DIALOG, a computer mediated procedure, was shown to improve outcomes in a European multi-centre trial. DIALOG structures the patient-clinician communication and makes it patient-centred, but does not guide clinicians as to how to respond to patients’ concerns. DIALOG has been further developed into DIALOG+, which uses advanced software and, additionally, provides a four step approach - based on a solution focused model - for addressing patients’ concerns. We designed a cluster randomised controlled trial to test the effectiveness of DIALOG+ in improving treatment outcomes of patients with psychosis in the community.

**Methods/design:**

Key workers are recruited from community mental health teams in East London and randomly allocated to either the intervention or control group. Out of their case loads, we identify patients with schizophrenia (F 20–29) and a moderate or lower level of subjective quality of life (MANSA score <5), who are treated according to the allocation of their key workers. Key workers in the intervention group are trained in using DIALOG+ and use it with each patient over a six-month period. Control patients rate their satisfaction with life and treatment on a tablet to control for the effect of regular ratings and the use of modern technology. We are recruiting up to 42 key workers to reach a total sample size of 180 patients. Clinical and social outcomes including costs are assessed after 3, 6 and 12 months. Primary outcome is subjective quality-of-life at 6 months.

**Discussion:**

The trial aims to evaluate the effectiveness of a novel intervention (DIALOG+) which uses modern technology to support routine patient-clinician meetings in community care, makes the communication patient centred and guides patients and clinicians to address concerns. DIALOG+ is a generic and widely applicable intervention. If shown as effective, it can be used to improve outcomes of community care on a large scale, ensuring that routine encounters are therapeutically effective. DIALOG+ can also be implemented across services at relatively low additional costs.

**Trial registration:**

Current Controlled Trials ISRCTN34757603

## Background

Approximately 1% of the population are affected by schizophrenia and related disorders with particularly high rates in urban areas [1]. Such disorders result in significant distress for patients and carers and account for a substantial societal burden. They also generate high costs to health care systems through the need for on-going intensive care and frequent hospitalisation, and to society at large caused by loss of employment of patients and frequently also carers [2,3].

Patients with severe forms of schizophrenia are now regularly cared for in the community. As a result of major reforms of mental health care since the 1970s and substantial additional investments in the last 10 years, multi-disciplinary community mental health care teams have been set up in various countries including the United Kingdom (UK) and provide on-going care. Every patient has a key worker (usually a nurse or social worker by background) who has regular meetings with the patient to assess their needs, engage them in treatment, and discuss different treatment options and co-ordinate care. Yet, the interaction in these meetings is based more on common sense than on evidence based methods.

Whilst research evidence suggests that a more positive patient-clinician relationship is associated with more favourable outcomes [4-7], there is no evidence based intervention to achieve a better therapeutic relationship in community mental health care. Also, until recently there was no evidence based method to structure the communication between patient and clinician in a way that would eventually lead to more favourable clinical outcomes [8].

DIALOG is the first method to structure the patient-clinician interaction in community mental health care that has been shown to be associated with more favourable long term outcomes in a randomised controlled trial [9]. In this computer-mediated intervention, patients are presented with fixed questions regarding their satisfaction with life and treatment and their needs for additional help. Subsequently, the ratings are displayed graphically and can be compared with ratings from previous meetings. In a cluster randomised controlled trial in six European countries, the DIALOG intervention was shown to be effective over a one year period. The intervention was associated with better subjective quality of life (SQOL), fewer unmet treatment needs and higher treatment satisfaction in patients with psychosis. The overall effect size was small. A stronger effect (medium effect size) was observed in the study site in the United Kingdom (East London) and in patients with more problematic baseline scores [10].

Following the trial, the National Institute for Health Research in the UK funded a programme to develop the intervention further both in terms of improving the technology and linking it with a simple psychological intervention informed by principles of Solution Focussed Therapy (SFT) and Cognitive Behavioural Therapy (CBT). The new intervention, combining the original intervention with a new 4-step approach to address problems raised by the patient, is called DIALOG+. The 4-step approach has been developed based on experiences with DIALOG in practice and involving experts in SFT and CBT. The four steps consist of exploring the reasons for the dissatisfaction or wish for additional help and identifying positive coping skills; a forward looking consideration of best hopes and/or small changes; a discussion of all options for helpful actions by the patient, the clinician or other people; and an agreement of actions to be taken. DIALOG+ is manualised, and a training programme has been designed with follow-up supervision for clinicians.

DIALOG+ differs considerably from the initial DIALOG intervention. DIALOG+ involves a new version of the software (DIALOG 2.0) on a new platform (a tablet), further structures clinicians’ behaviour according to the DIALOG+ manual, and involves training of clinicians. DIALOG+ aims to provide a way to deal with the specific concerns raised by the patient and, hence, equip the clinician as well as the patient with a method to explore and deal with problems.

Furthermore, the DIALOG+ intervention is more intensive. It is being delivered at least once a month over a six month period, rather than every two months over a one-year period, as in the original trial. We have included a wider range of patients, as opposed to those with persistent disorders who had been in care for an average of 15 years in the previous study. Finally, we are comparing the intervention with a more defined control condition than treatment as usual. This will help to control for the effect of the implementation of an electronic device in the clinical setting and the assessment of satisfaction; without providing feedback, expanding on problematic domains or exploring needs for additional help. All of these differences justify a new trial testing the effectiveness of DIALOG+.

Against this background, the trial aims to test whether the regular use of the DIALOG+ intervention over a six month period improves patients’ outcomes, and if it is cost-effective.

## Methods

### Study design

In a pragmatic cluster randomised controlled trial key workers and their patients are randomly allocated to the experimental condition using DIALOG+ or the control condition with regular ratings of satisfaction using the DIALOG Scale on a tablet. Both conditions are in addition to treatment as usual. There are four data collection points: baseline, and 3, 6 and 12 month follow-ups.

Key workers are recruited from Community Mental Health Teams in East London (East London NHS Foundation Trust). We aim to recruit a maximum of seven patients per key worker. If necessary, the patients to approach for participation are randomly selected from the pool of eligible patients on a key worker’s caseload. However, this is rare, and usually we recruit fewer than seven patients per key worker. Once all consenting patients (per key worker) meeting the inclusion criteria have been recruited, the key worker – and their patients – are randomly allocated to either the intervention or control condition. Clustering of clinicians prevents contamination effects in the study. Randomisation is carried out via e-mail by an independent statistician at the Pragmatic Clinical Trials Unit of Queen Mary, University of London. The unit of randomisation is the key worker using randomly permuted blocks with variable block sizes and equal allocation ratio.

Key workers use the intervention with their patients for a period of six months. Midway through receiving the intervention (3 months), at the end of the intervention period (6 months), and after a 6 month follow-up period patients are interviewed by researchers and outcomes assessed. These assessments are conducted on NHS sites or in the community.

Researchers assessing the outcomes are blinded to the allocation of the patients. If blinding cannot be maintained, we put researchers working at the Unit for Social and Community Psychiatry at Queen Mary, University of London (who are independent of the core research team) into contact with patients via telephone for conducting the assessment of the primary outcome and, as far as feasible, secondary outcomes. In such cases, patients are asked not to discuss the treatment they received for the duration of the call.

### Planned interventions

Key workers and their patients in the experimental condition are instructed to use DIALOG+ at least once per month over a six month period, although this may vary due to the practical organisation of care. DIALOG+ is used as defined in the accompanying manual, and the DIALOG 2.0 software runs on a tablet. All key workers in the experimental group are trained in one session before beginning the intervention. The training is provided by two experienced clinicians who are independent of the research study team (both qualified in psychological treatments), with the number of training sessions divided between the two. For practical reasons, the majority of training is carried out one to one. During the training session, key workers are provided with reading material and the DIALOG+ manual, have the procedure demonstrated to them, participate in a role-play exercise, and have the opportunity to watch videos. For each key worker, the first DIALOG+ session with a patient is audio-recorded and feedback is provided by their trainer via a follow-up session. A subsequent DIALOG+ session is also audio-recorded to provide further feedback. Throughout the duration of the study key workers can contact the trainers for further sessions for support and advice at any time.

Key workers and patients in the experimental group may decide to continue with DIALOG+ after the end of the six month intervention. This will be documented and considered in the analysis of outcomes after the follow-up period.

The control condition includes treatment as usual plus a defined intervention that also involves the use of a tablet and an assessment of the patient’s satisfaction. The patients use the device to rate their satisfaction on the 11 domains of the DIALOG scale. The intervention is delivered with the same frequency as DIALOG+, with such assessments being undertaken after a routine meeting. The key workers are instructed to demonstrate the software to the patients prior to their first time completing the scale. Patients need to complete the scale alone without the key worker’s help, and patients and key workers are not to discuss the ratings. This is intended to control for the effect of the implementation of an electronic device in the clinical setting and the regular assessment of satisfaction.

### Inclusion and exclusion criteria

To reflect the pragmatic nature of the trial, there are wide inclusion criteria and very few exclusion criteria for both clinicians and patients. The inclusion criteria for patients are wider than in the original DIALOG trial to test whether patients with more acute disorders, who have not been in care for many years and whose treatment in secondary services may not continue for another year, can also be recruited and benefit from the intervention.

#### Key workers

Clinicians are key workers currently employed in the East London NHS Foundation Trust, working in community mental health teams and meeting the following criteria:

• Professional qualification as a clinician (nurse, social worker, psychologist, occupational therapist, doctor);

• More than 6 months experience of working in community mental health care;

• Working as care coordinator.

Clinicians are excluded if they have to attend a scheduled training course of more than two weeks’ time during the study period and will not continue their care-coordinating role whilst attending training.

#### Patients

We recruit patients in the East London NHS Foundation Trust, currently treated in a community mental health care team, fulfilling the following criteria:

• Treatment in a community mental health care team for at least one month;

• No planned discharge for the next six months;

• Clinical diagnosis of schizophrenia or a related disorder (F20-29);

• Age between 18 and 65 years;

• A mean score of lower than 5 on the MANSA;

• Capacity to give informed consent.

#### Exclusion criteria

• Insufficient command of the English language for conducting meetings in English and filling in the assessment instruments of outcomes;

• A mean score of 5 or more on the MANSA;

• Learning difficulties.

### Outcome measures

Outcomes are assessed at baseline and after 3, 6, and 12 months.

The primary outcome is subjective quality of life, measured on the Manchester Short Assessment of Quality of Life (MANSA) (as in the original trial testing DIALOG) [11]

Secondary outcomes for the trial are:

• Recovery as measured on the severity parts of each of the 24 items of the CHOICE scale [12] (satisfaction, i.e. the second part of each item, is already assessed on the MANSA)

• Objective Social Outcomes (SIX) [13], also assessed using the MANSA (sections 2 and 3), at 6 and 12 months only

• Social contacts in the last week in a structured interview

• Treatment Satisfaction on the Client Satisfaction Questionnaire (CSQ-8) [14]

• The Therapeutic Relationship on the Scale for Assessing Therapeutic Relationships in community mental health care (patient and clinician versions) [15]

• Needs on the Camberwell Assessment of Need Short Appraisal Schedule, patient-rated version [16]

• Self-Efficacy on the General Self-Efficacy Scale [17]

• Psychopathological Symptoms on the Brief Psychiatric Rating Scale [18]

• Costs of care, assessed on the Client Service Receipt Inventory [19], i.e. data on the use and frequency of inpatient care, outpatient care (face to face meetings), and other health services which are obtained from case notes, electronic databases and patients themselves.

• Mental well-being on the Warwick-Edinburgh Mental Well-Being Scale (WEMWBS) [20].

All outcomes are assessed at baseline and after 3, 6, and 12 months.

### Qualitative assessments

For assessing the feasibility of the intervention, adherence to the manual and its effects in the patient-clinician meetings, each patient-clinician pair in the intervention group has one of their meetings video-taped (or when unpractical audio-taped) and analysed. In the control group, we do the same for 20% of the sample. Adherence to manual in the intervention group is assessed against the manual for DIALOG+, using an adherence scale developed by the research team.

The software records automatically what domains were explicitly discussed in DIALOG+ and what actions were agreed. This information is synchronised to a server that un-blinded members of the research team have access to.

At the end of the intervention, separate focus groups will be conducted and audio-taped with patients and clinicians in the intervention group to obtain their experiences and suggestions for improvements.

### Statistical analysis

The primary analysis of subjective quality of life as measured on the MANSA will be conducted using a generalised linear model with a fixed effect for treatment and baseline MANSA score and a random effect for key worker to account for clustering.

Secondary outcomes will be analysed using a similar approach, with adjustment for corresponding baseline values.

Sensitivity analyses will be performed to explore the impact of missing data on our conclusions.

All analysis will be conducted under the intention to treat principle and significance testing will be at the 5% level (2-sided). A full analysis plan will be developed and agreed prior to any analysis or unblinding of the data. Results will be presented to follow the recommendations given in the CONSORT statement extension for the reporting of cluster randomised trials.

### Economic evaluation

The cost of the DIALOG and DIALOG+ interventions are expected to be low and will mainly comprise any extra time spent with the patient to administer the intervention. There will though be costs associated with (i) refining the procedure and developing DIALOG+ (but when apportioned across all patients who might receive the intervention these will be minimal), and (ii) training key workers in their use. A nominal developmental cost per patient will be included and resources and time required for training will be measured and costed. Total costs will be calculated by combining the intervention costs with those associated with the use of other services. Cost differences will be analysed using bootstrap methods due to the expected skewness in the data distributions. Costs will be combined with the primary outcome measure in the form of a cost-effectiveness analysis. If costs are lower for one group than another and outomes are better then it will be ‘dominant’ in terms of cost-effectiveness. If costs are higher and outcomes are better then an incremental cost-effectiveness ratio will indicate the extra costs incurred to produce an extra unit-improvement in quality of life. Uncertainty around these estimates will be analysed using cost-effectiveness planes and cost-effectiveness acceptability curves [21].

### Qualitative analysis

Videotapes will be analysed according to specific criteria (using a purposely developed adherence scale) for adherence to the manual. Audiotapes from focus groups will be analysed using thematic analysis.

### Proposed sample size

We aim to recruit 42 key workers in total; with each key worker providing an average of four to five patients, providing a total of 180 patients.

Assuming a practically negligible cluster effect (as in the original trial) the sample size will be sufficient to detect a medium effect size of 0.5 (Cohen’s D) with 80% power at the 5% significance level (2-sided). A slightly higher effect of 0.57 will be detected with 90% power.

The effect size of 0.5 would require that at the end of the intervention patients in the experimental condition rate their satisfaction with at least 3 out of 12 life domains in the MANSA at least one point higher than patients in the control condition.

This sample size allows for a cluster attrition rate of 6 key workers (30 patients) and a patient dropout rate of less than 10%.

### Recruiting participants and obtaining informed consent

Clinicians working in Community Mental Health Teams in the East London boroughs of Hackney, Newham and Tower Hamlets are approached by the managers of East London NHS Foundation Trust and asked to participate in the study. Written informed consent to participate is obtained from individual key workers.

Key workers, supported by research and administrative staff, identify patients on their caseloads fulfilling the inclusion criteria, and then ask suitable patients for consent to be approached by a researcher. If the answer is yes, a researcher arranges a meeting with the patient to provide an information sheet and to discuss the project in full. At the meeting, the researcher goes through the information sheet, explains the study, obtains written informed consent, and establishes all inclusion criteria.

Patients are informed through their consent forms that they are free to withdraw from the study at any time, without giving reason and without any negative consequences. This will be emphasised by the researcher in all discussions explaining the study and prior to taking informed consent.

As part of this process we obtain consent for one of the patient’s sessions with their key worker to be video recorded in order to assess adherence to the procedure, and for patients to attend an audio-recorded focus group at the end of their intervention period to discuss their experiences with other patients. This is optional for patients, and if they do not wish to be video recorded or take part in the focus group this will not impact on their participation in the study.

### Ethics approval

The study has been approved by the London-Stanmore National Research Ethics Service Committee (REC reference 12/LO/1145). All data will be stored in line with the Data Protection Act. All audio and video recorded data will be encrypted and password protected.

### Trial steering committee and data monitoring and ethics committee

Both a Trial Steering Committee and a Data Monitoring and Ethics Committee have been set up. No formal interim efficacy analyses have been planned.

## Discussion

At present, large numbers of patients with psychosis regularly meet key workers in community mental health teams, but the patient-clinician interactions are not guided by evidenced based principles. A method to make these interactions more effective will not be a specialist programme for a small number of patients, but a generic method that can be utilised in routine care throughout the NHS and potentially in other countries with similar services providing care in the community for patient with psychosis (DIALOG+). It does not require setting up new services or restructuring of organisations. It can be implemented at relatively low costs, particularly as it will not require extensive training of clinical staff, and can benefit tens of thousands of patients in the NHS at the same time. Thus, even small health and social gains for individual patients will add up to substantial public health effects. This also applies to potential cost savings. The FOCUS study (in which patients’ outcomes were assessed by researchers and fed back to clinicians without structuring clinician-patient communication) suggested annual cost savings of regular outcome data feedback (which is also provided in DIALOG+) equivalent to £5172 per patient through reduced bed use [22]. If replicated for only 20% of patients with schizophrenia and related disorders in community care in the NHS, the savings would exceed £100 million every year.

The procedure of DIALOG+ will also provide regular outcome data, i.e. patients’ ratings of satisfaction with life, treatment satisfaction and needs for further care. This data can be used to evaluate services on a local, regional and national level. So far, attempts to establish outcome assessment in routine community mental health care have largely failed, partly because it is difficult to motivate clinicians and patients to rate and enter outcome data on a regular basis. DIALOG+ provides a method to generate such data in way that is meaningful to clinicians and patients, and is likely to facilitate routine outcome assessment in secondary mental health services in the NHS.

The trial pursues the ambitious aim of testing a new intervention that can be rolled out widely and at low costs, and – if successful - ensures that routine patient-clinician meetings in the community are therapeutically effective. The intervention is based on well-established models of psychological treatments, in particular on solution focused therapy, but has been specified so that it is easy to train, enables clinicians to bring in existing therapeutic skills and uses modern technologies. The trial involves various teams across East London and is pragmatic in terms of being implemented in routine care.

## Competing interests

There are no competing interests for any of the authors.

## Authors’ contributions

SP coordinated and produced the initial outline of the application, is chief investigator for the ‘EPOS’ trial, and was involved in the development of the protocol and preparation for publication. LK contributed to the preparation of the protocol for publication. EG draft of proposal, design and development of the protocol. PM prepared the plans for economic analysis. DK contributed to the design and development of the protocol. CR contributed to the methodology and analysis plan. RM draft of proposal, design and development of the protocol. All authors have read and approved the final manuscript.

## Pre-publication history

The pre-publication history for this paper can be accessed here:

http://www.biomedcentral.com/1471-244X/13/173/prepub

## References

[B1] Lloyd T Kennedy N Fearon P Kirkbride J Mallett R Leff J Incidence of bipolar affective disorder in three UK cities: results from the AESOP study Brit J Psychiat 2005 186 126 131 10.1192/bjp.186.2.126 15684235

[B2] Gupta RD Guest JF Annual cost of bipolar disorder to UK society Brit J Psychiat 2002 180 227 233 10.1192/bjp.180.3.227 11872515

[B3] National Institute for Health and Care Excellence The clinical effectiveness and cost effectiveness of newer atypical antipsychotic drugs for schizophrenia. TA43 2002 London: National Institute for Health and Care Excellence

[B4] Klinkenberg WD Calsyn RJ Morse GA The helping alliance in case management for homeless persons with severe mental illness Community Ment Hlt J 1998 34 569 578 10.1023/A:1018758917277 9833198

[B5] McCabe R Priebe S The therapeutic relationship in the treatment of severe mental illness: a review of methods and findings Int J Soc Psychiatr 2004 50 115 128 10.1177/0020764004040959 15293429

[B6] Priebe S Gruyters T The role of the helping alliance in psychiatric community care. A prospective study J Nerv Ment Dis 1993 181 552 557 10.1097/00005053-199309000-00004 8245923

[B7] Tattan T Tarrier N The expressed emotion of case managers of the seriously mentally ill: the influence of expressed emotion on clinical outcomes Psychol Med 2000 30 195 204 10.1017/S0033291799001579 10722190

[B8] Priebe S McCabe R The therapeutic relationship in psychiatric settings Acta Psychiat Scand 2006 429 69 72 10.1111/j.1600-0447.2005.00721.x 16445486

[B9] Priebe S McCabe R Bullenkamp J Structured patient-clinician communication and one-year outcome in community mental health care: a cluster randomised controlled trial Brit J Psychiat 2007 191 420 426 10.1192/bjp.bp.107.036939 17978322

[B10] Van den Brink R Wiersma D Wolters K Bullenkamp J Hansson L Lauber C Martinez-Leal R McCabe R Rossler W Salize H Svensson B Torres-Gonzales F Priebe S Non-uniform effectiveness of structured patient-clinician communication in community mental healthcare: an international comparison (DIALOG) Soc Psych Psychi Epid 2011 46 685 693 10.1007/s00127-010-0235-x 20490455

[B11] Priebe S Huxley P Knight S Evans S Application and results of the Manchester short assessment of quality of life (MANSA) Int J Soc Psychiatr 1999 45 7 12 10.1177/002076409904500102 10443245

[B12] Greenwood KE Sweeney A Williams S Garety P Kuipers E Scott J Peters E Choice of outcome in CBT for psychosis (CHOICE): the development of a new service user–led outcome measure of CBT for psychosis Schizophrenia Bull 2010 36 126 135 10.1093/schbul/sbp117 PMC2800145 19880823

[B13] Priebe S Watzke S Hansson L Burns T Objective social outcomes index (SIX): a method to summarise objective indicators of social outcomes in mental health care Acta Psychiat Scand 2008 118 57 63 10.1111/j.1600-0447.2008.01217.x 18582348

[B14] Nguyen TD Attkisson CC Stegner BL Assessment of patient satisfaction: development and refinement of a service evaluation questionnaire Eval Program Plann 1983 6 299 313 10.1016/0149-7189(83)90010-1 10267258

[B15] McGuire-Snieckus R McCabe R Catty J Hansson L Priebe S A new scale to assess the therapeutic relationship in community mental health care: STAR Psychol Med 2007 37 85 95 10.1017/S0033291706009299 17094819

[B16] Phelan M Slade M Thornicroft G Parkman S The Camberwell assessment of need (CAN): comparison of assessments by staff and patients of the needs of the severely mentally ill Social Psych Psych Epid 1996 31 109 113 10.1007/BF00785756 8766455

[B17] Schwarzer R Jerusalem M Weinman J, Wright S, Windsor JM General self-efficacy scale Measures in health psychology: a user’s portfolio. Causal and control beliefs 1995 UK: Nfer-Nelson 35 37

[B18] Overall JE Gorham DR The brief psychiatric rating scale Psychol Rep 1962 10 799 812 10.2466/pr0.1962.10.3.799

[B19] Beecham J Knapp M Thornicroft G Costing psychiatric interventions Measuring mental health needs 2001 2 London, UK: Gaskell 220 224

[B20] Tennant R Hiller L Fishwick R Platt S Joseph S Weich S Parkinson J Secker J Stewart-Brown S The Warwick-Edinburgh mental well-being scale (WEMWBS): development and UK validation Health Qual Life Outcomes 2007 5 63 10.1186/1477-7525-5-63 18042300 PMC2222612

[B21] McCrone P Dhanasiri S Patel A Knapp M Paying the price: the cost of mental health care in England to 2026 2008 London: King’s Fund

[B22] Slade M McCrone P Kuipers E Leese M Cahill S Parabiaghi A Use of standardised outcome measures in adult mental health services Brit J Psychiat 2006 189 330 336 10.1192/bjp.bp.105.015412 17012656

